# Development of portable nanofocusing optics for X-ray free-electron laser pulses

**DOI:** 10.1107/S1600577525002279

**Published:** 2025-04-09

**Authors:** Yuichi Inubushi, Gota Yamaguchi, Jumpei Yamada, Yuya Kubota, Ichiro Inoue, Taito Osaka, Toshinori Yabuuchi, Kensuke Tono, Makina Yabashi

**Affiliations:** a Japan Synchrotron Radiation Research Institute 1-1-1 Kouto, Sayo-cho Sayo-gun Hyogo679-5198 Japan; b RIKEN SPring-8 Center, 1-1-1 Kouto, Sayo-cho, Sayo-gun, Hyogo679-5148, Japan; c Graduate School of Engineering Osaka University 2-1 Yamada-oka Suita Osaka565-0871 Japan; RIKEN SPring-8 Center, Japan

**Keywords:** X-ray free-electron lasers, nanofocusing, *K*α lasers

## Abstract

We present the development of a portable and compact nanofocusing system utilizing Kirkpatrick–Baez optics for X-ray free-electron lasers (XFELs). The system has a total length of merely 340 mm from the initial elliptical mirror to the focal point. With this setup, we achieve focusing capabilities reaching dimensions as small as 150 nm horizontally and 220 nm vertically, resulting in an intense beam with an intensity of 2.5 × 10^19^ W cm^−2^ for a 10 keV XFEL with a pulse energy of 110 µJ. As a demonstration of its applicability in X-ray nonlinear optics, we successfully observed an 8.64 keV Zn *K*α laser by irradiating a Zn foil with the focused XFEL beam. The attained photon energy is currently the highest photon energy for amplified spontaneous emission via bound–bound transitions of electrons in the world. We anticipate that this portable and compact nanofocusing system will pave the way for new frontiers in X-ray nonlinear optics and high-energy density science, owing to its adaptability to various experimental conditions.

## Abstract 2

A portable and compact nanofocusing system was developed for X-ray free-electron lasers with the ability to generate Zn *K*α lasers with the world’s highest photon energies on amplified spontaneous emission.

## 1. Introduction

The recent advancements in X-ray free-electron lasers (XFELs) (Emma *et al.*, 2010 ▸; Ishikawa *et al.*, 2012 ▸) have made it possible to generate brilliant femtosecond X-ray pulses with ultra-high spatial and temporal resolution. XFEL facilities have played an important role in opening up new fields of X-ray science (Chapman *et al.*, 2011 ▸; Suga *et al.*, 2015 ▸; Kim *et al.*, 2020 ▸). At SACLA, the implementation of high-intensity XFEL pulses generated with nanofocusing optics (Mimura *et al.*, 2014 ▸; Yumoto *et al.*, 2020 ▸) has opened the way to explore new scientific frontiers, including nonlinear X-ray optics (Tamasaku *et al.*, 2014 ▸; Yoneda *et al.*, 2014 ▸, 2015 ▸; Inoue *et al.*, 2016 ▸; Zhang *et al.*, 2022 ▸; Kubota & Tasamaku, 2023 ▸). Typically, nanofocusing optics have relied on Kirkpatrick–Baez (KB) optics, which consist of two elliptical mirrors (Mimura *et al.*, 2014 ▸; Yumoto *et al.*, 2020 ▸). These mirrors employ grazing incidence optics and require lengths of several hundred millimetres to accommodate the grazing incidence footprint of the X-ray beam. While these systems have provided excellent focusing capabilities, their installation in dedicated experimental hutches has limited the range of experiments that can be conducted due to instrument availability constraints. However, the development of a portable nanofocusing system makes it possible to conduct experiments across various experimental setups, combining with dedicated instruments such as synchronized optical laser systems and large area detectors. In this article, we report on the development of a portable compact nanofocusing system and demonstrate its capabilities through the generation of XFEL-pumped Zn *K*α laser pulses with the world’s highest photon energy using bound–bound electron transitions.

## 2. Design of a portable nanofocusing system

The diffraction-limit focal size ϕ of focusing optics for the specific wavelength λ is determined by the focal length *f* and the spatial acceptance *D*, as shown by
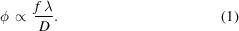
Although equation (1) appears to indicate that a large *D* is beneficial to obtain a small focal spot size ϕ, *D* should be optimized to match the size of the incident XFEL beam to the mirror. Therefore, a shorter focal length *f* is required to achieve a smaller focal size ϕ. In the X-ray region, a total reflection mirror for grazing incidence is utilized as focusing optics. In this case, the focal length *f* should be longer than the half of the effective length *L* of the focusing mirror. Thus, a compact mirror is required to obtain shorter *f*, supporting the enhancement of portability of the focusing system. Additionally, owing to the shorter *f*, both deviations of focused X-rays on the focusing plane due to the angle error of the grazing incidence and influence by a figure error of the mirror fabrication are suppressed (Shimamura *et al.*, 2024 ▸). These enhancements of stability are important advantages for a compact portable nanofocusing system. However, there are some disadvantages due to the compactness of the focusing optics. One such drawback is the difficulty in obtaining a long working distance (WD), defined as the distance from the downstream edge of the second mirror substrate to the focal point. This is due to the small *f*, as *f* is the sum of the WD and half the mirror substrate length for grazing incidence optics. Therefore, it is important to design considering the constraints of experimental conditions. Another issue is the difficulty in utilizing a large spatial acceptance *D*, which is represented by the following equation using *L* and the incident grazing angle θ,
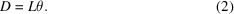
For portable mirror systems, it is effective to use a large θ to obtain sufficient *D* for the XFEL beam size. In this case, a mirror surface coating is required to suppress the decrease of the reflectivity.

**Figure 1:**
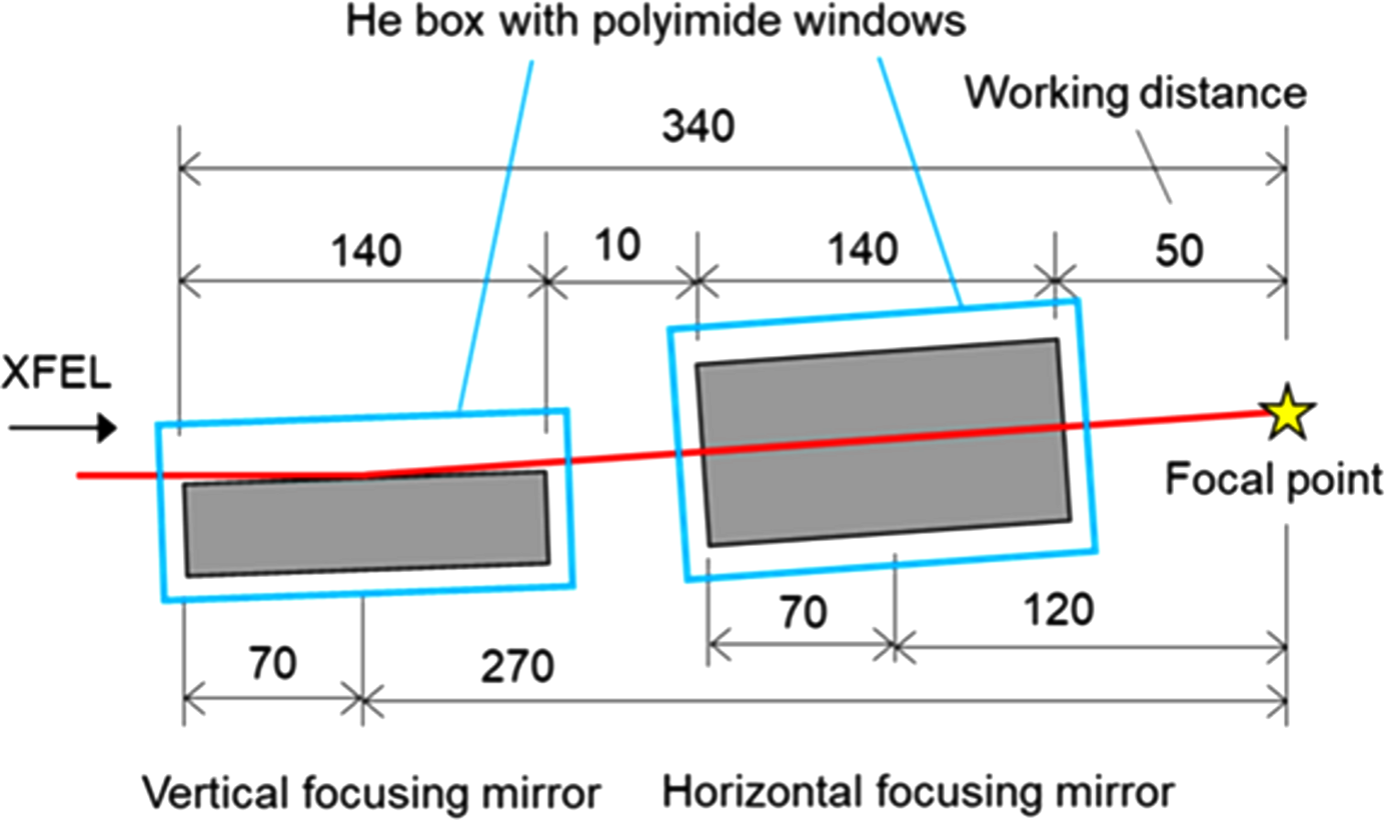
Schematic image of a side view of the nanofocusing optics. The unit of length is millimetres. Each elliptical mirror is enclosed within a helium-filled box with polyimide windows. The grazing angles for both mirrors are set to 5 mrad. The total length from the upstream edge of the vertical focusing mirror to the focal point measures 340 mm.

We have designed a portable nanofocusing system based on KB optics with two elliptical mirrors, considering the conditions mentioned above. The optical parameters for each mirror are summarized in Table 1 ▸. The focal length for the vertical focusing mirror is 270 mm, and for the horizontal focusing mirror it is 120 mm. The large grazing angle of 5 mrad enables a spatial acceptance of 650 µm, even for the short effective mirror length of 130 mm. To maintain a reflectivity of ∼80%, the vertical and horizontal mirrors are coated with ruthenium (Ru) and platinum (Pt), respectively. The effective energy range of the focusing system is below 11.5 keV due to the Pt *L*-absorption edge. The mirrors were manufactured by the JTEC Corporation. Using these mirrors, we realized a compact nanofocusing system with a total length of 340 mm from the upstream edge of the first elliptical mirror to the focal point, including a WD of 50 mm, as shown in Fig. 1 ▸.

## 3. Focusing and demonstration of application

**Figure 2:**
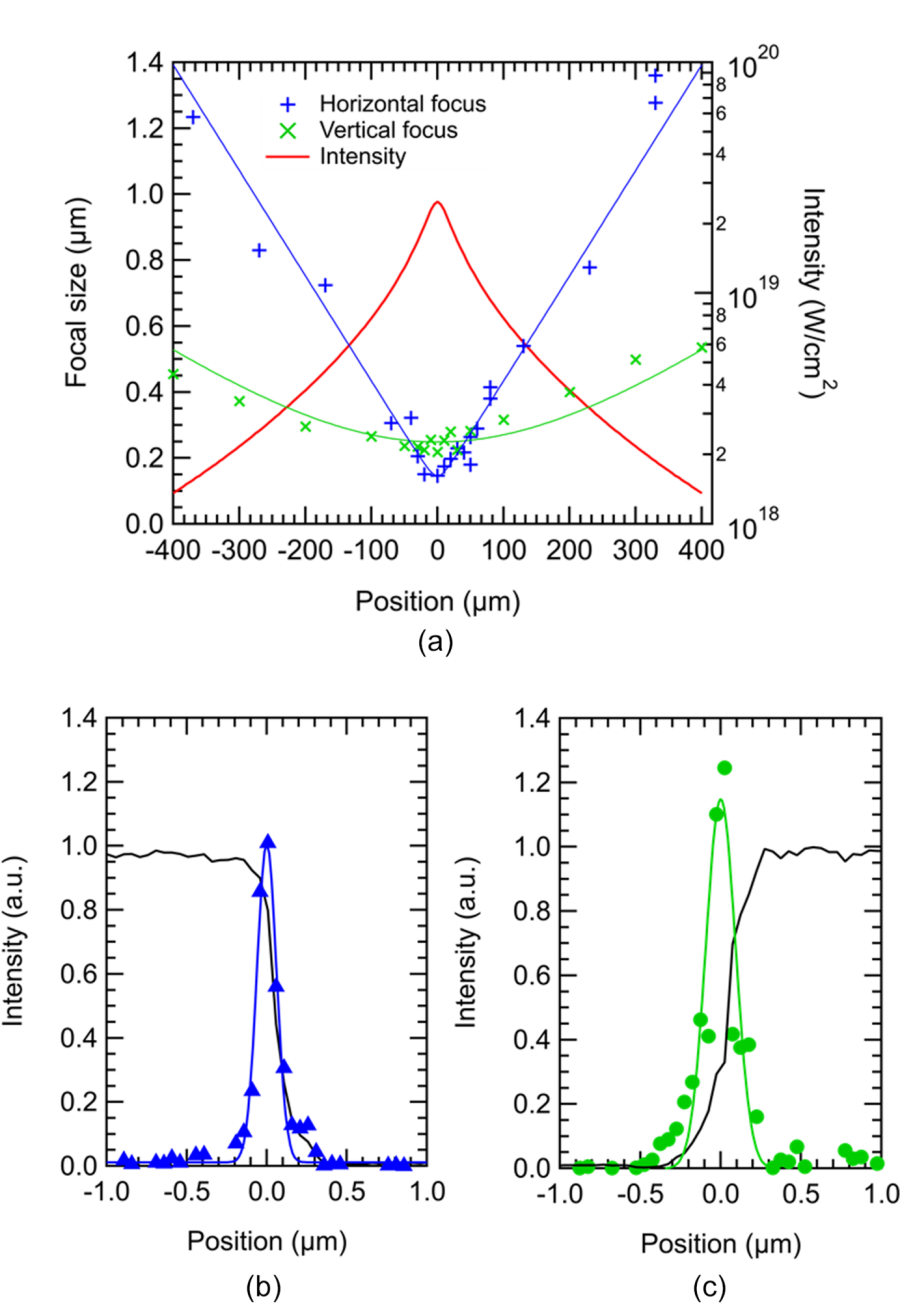
(*a*) Beam sizes and intensities depending on the position along the XFEL axis. The positive direction of the position is upstream. The highest intensity was obtained to be 2.5 × 10^19^ W cm^−2^. Also, results of the knife-edge scanning method for (*b*) horizontal and (*c*) vertical directions. The focal sizes, derived using the differential results of raw data, were determined to be 150 nm (horizontal) and 220 nm (vertical).

The focusing experiment was conducted using XFEL pulses with a photon energy of 10 keV at SACLA BL2 EH3. Typically, a pulse energy of 110 µJ was reached at the focal point, resulting in a throughput from the exit of the undulators to the focal point of 25%. Fig. 2 ▸(*a*) illustrates the horizontal and vertical beam sizes depending on the position along the XFEL beam axis. The minimum beam sizes were measured to be 150 nm and 220 nm in full width at half-maximum (FWHM) for the horizontal and vertical directions, respectively, as depicted in Figs. 2 ▸(*b*) and 2 ▸(*c*). Additionally, Fig. 2 ▸(*a*) includes a calculation of XFEL intensity depending on the position, utilizing the beam sizes, pulse energy and a pulse duration of 8 fs (Inubushi *et al.*, 2012 ▸, 2017 ▸). The highest intensity achieved was 2.5 × 10^19^ W cm^−2^.

To demonstrate the application of X-ray nonlinear optics using the portable focusing system, we conducted an experiment to generate XFEL-pumped Zn *K*α laser pulses. When the intensity of X-rays with higher photon energy than the*K*-absorption edge of a sample material is extremely high, population inversion can be created by ionization of a *K*-shell electron. Then, the spontaneous emission of the *K*α line is amplified via the stimulated emission process and becomes a laser (Duguay & Rentzepis, 1967 ▸; Kapteyn, 1992 ▸; Zhao *et al.*, 2008 ▸; Rohringer & London, 2009 ▸; Rohringer *et al.*, 2012 ▸; Yoneda *et al.*, 2015 ▸). However, the *K*-shell core hole is rapidly filled through Auger and radiative decay processes. Moreover, as ionization progresses due to electron collisions, the transition energy shifts, making lasing difficult. A high-intensity XFEL is essential to create a population inversion within the shorter period before these decay processes occur. To achieve amplified spontaneous emission (ASE) laser oscillation at high photon energies, materials with high atomic numbers are necessary; however, the lifetime of the *K*-shell core hole decreases as the atomic number increases. Consequently, the higher the photon energy of the ASE laser oscillation, the greater the required intensity. Specifically, the pump intensity needed for *K*α laser oscillation scales with the fourth power of the photon energy (Yoneda *et al.*, 2015 ▸). Thus, an experiment toward lasing of the ASE laser serves as a valuable benchmark for the nanofocusing system.

**Figure 3:**
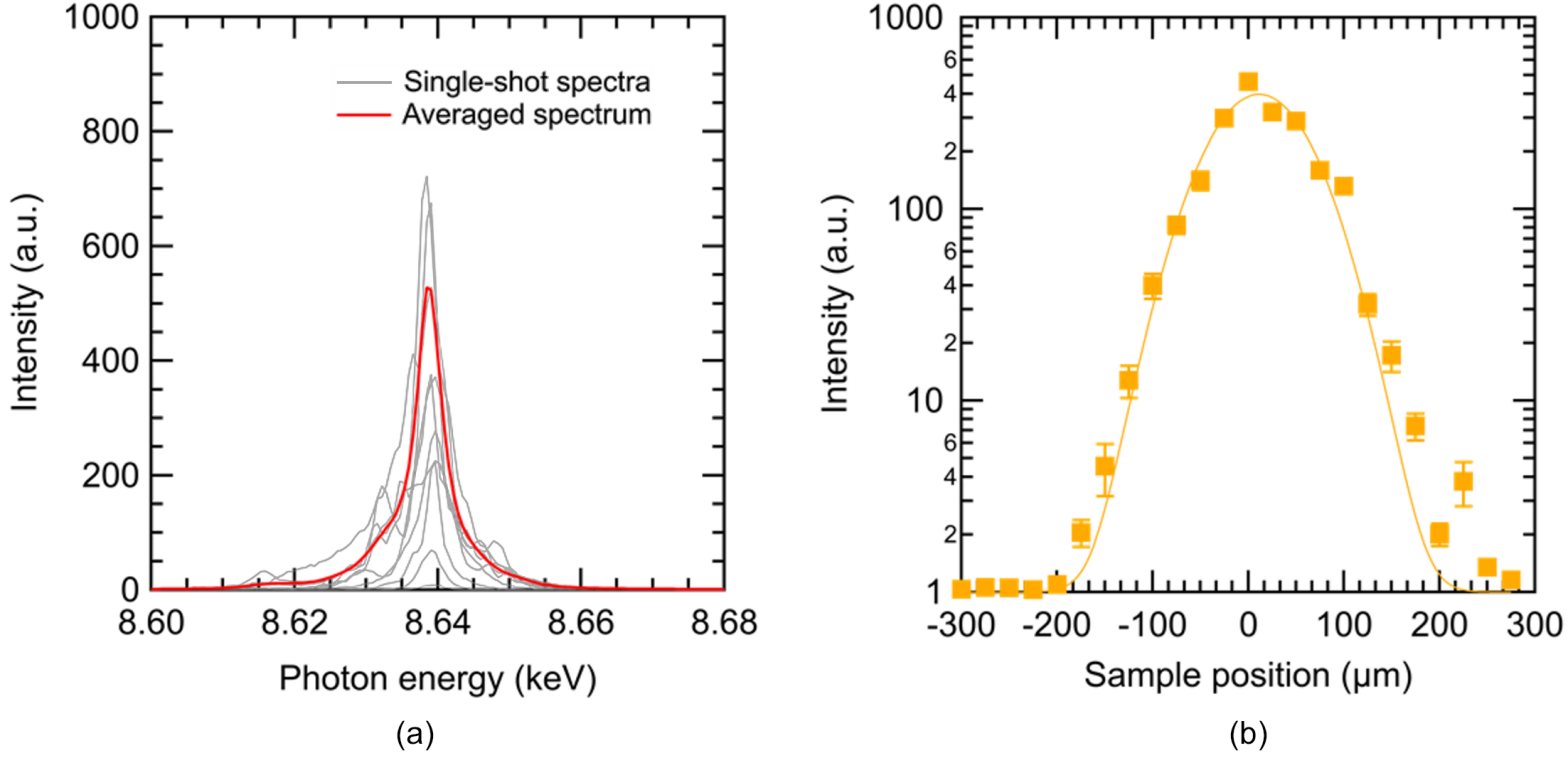
(*a*) Spectra of a Zn *K*α laser measured at the position of the highest intensity. The gray curves represent some single-shot spectra, while the red curve displays a 175-pulse-averaged spectrum. (*b*) The averaged peak intensities of the Zn *K*α laser as a function of the position of the Zn foil.

The experiment was also conducted at SACLA BL2 EH3, where 10 keV XFEL pulses were focused on a 25 µm-thick Zn foil. Since the *K*-absorption edge of Zn is 9.66 keV, the 10 keV XFEL could ionize a *K*-shell electron. The Zn foil, placed in an air environment, was moved shot-by-shot for irradiation on a fresh surface. The Zn *K*α laser was measured with a single-shot X-ray spectrometer consisting of a silicon(111) analyzer crystal and a multi-port charge-coupled device detector (Kameshima *et al.*, 2014 ▸). In this setup, the energy dispersion plane was vertical, and the observation range and pixel resolution were 90 eV and 630 meV, respectively. Fig. 3 ▸(*a*) displays the spectra measured at the position with the highest XFEL intensity. Strong peaks at 8.64 keV, corresponding to the Zn *K*α line, are evident. The red line represents a 175-pulses-averaged spectrum, while the gray lines depict some single-shot spectra. The averaged peak intensities as a function of the position of the Zn foil are shown in Fig. 3 ▸(*b*). Referring also to Fig. 2 ▸(*a*), a nonlinear increase of the detected *K*α signal as a function of increasing XFEL intensity is observed. These results lead to the conclusion that the Zn *K*α laser was successfully generated in the experiment. The observed Zn *K*α laser of 8.64 keV represents the highest photon energy of an X-ray laser generated via the ASE process by a bound–bound transition of electrons, surpassing previous reports of *K*α lasers using Ne (Rohringer *et al.*, 2012 ▸), Mn (Kroll *et al.*, 2018 ▸, 2020 ▸; Zhang *et al.*, 2022 ▸; Doyle *et al.*, 2023 ▸) and Cu (Yoneda *et al.*, 2015 ▸). Additionally, from Figs. 2 ▸(*a*) and 3 ▸(*b*), it was determined that the threshold intensity for the Zn *K*α laser is ∼4 × 10^18^ W cm^−2^.

## 4. Summary and future perspectives

We developed a portable compact nanofocusing system based on KB optics for XFEL pulses, achieving a focal size of 150 nm × 220 nm (FWHM) in the horizontal and vertical directions. Utilizing focused XFEL pulses, we successfully generated a Zn *K*α laser at 8.64 keV. These results highlight the potential of our new nanofocusing system, characterized by compactness and portability, to contribute to advancements in scientific fields such as nonlinear X-ray optics and high-energy density science. Moreover, we anticipate that novel scientific achievements will emerge through the synergistic combination of various experimental instruments.

## Figures and Tables

**Table 1:** Optical parameters of the focusing mirrors

	Vertical focusing mirror	Horizontal focusing mirror
Surface profile	Elliptical cylinder	Elliptical cylinder
Substrate material	Synthetic quartz	Synthetic quartz
Surface coating	Ruthenium	Platinum
Mirror substrate size	140 mm × 50 mm × 30 mm	140 mm × 50 mm × 30 mm
Grazing angle on optical axis	5.0 mrad	5.0 mrad
Focal length	270 mm	120 mm
Effective mirror length	130 mm	130 mm
Spatial acceptance	650 µm	650 µm
Numerical aperture	0.0012	0.0027
Figure error†	0.18 nm	0.22 nm
Surface roughness†	0.15 nm	0.10 nm

† In root mean square.
